# Inferring Transcriptional Interactions by the Optimal Integration of ChIP-chip and Knock-out Data

**DOI:** 10.4137/bbi.s3445

**Published:** 2009-10-21

**Authors:** Haoyu Cheng, Lihua Jiang, Maoying Wu, Qi Liu

**Affiliations:** School of Life Sciences and Biotechnology, Shanghai Jiao Tong University, Shanghai 200240, China. Email: liuqi@sjtu.edu.cn

**Keywords:** regulatory interaction, transcription factor, ChIP, knock-out data, P-value threshold, hypergeometric distribution, cooperativity

## Abstract

How to combine heterogeneous data sources for reliable prediction of transcriptional regulation is a challenge. Here we present an easy but powerful method to integrate Chromatin immunoprecipitation (ChIP)-chip and knock-out data. Since these two types of data provide complementary (physical and functional) information about transcription, the method combining them is expected to achieve high detection rates and very low false positive rates. We try to seek the optimal integration of these two data using hyper-geometric distribution. We evaluate our method on yeast data and compare our predictions with YEASTRACT, high-quality ChIP-chip data, and literature. The results show that even using low-quality ChIP-chip data, our method uncovers more relations than those inferred before from high-quality data. Furthermore our method achieves a low false positive rate. We find experimental and computational evidence in literature for most transcription factor (TF)-gene relations uncovered by our method.

## Introduction

The dynamic program that a cell utilizes in response to internal and external stimuli is carried out through coordinated action of many genes and proteins. Transcriptional regulation plays an important role in the program. Thus unraveling transcriptional interactions is critical to our understanding of the complex regulation mechanisms.

Recent advances in high-throughput DNA micro-arrays and chromatin immunoprecipitation (ChIP) assays have provided us with an unprecedented amount of information about transcriptional regulation on a genomic scale. Gene expression profiles under various conditions are the key data source for inferring transcriptional relations. Some researchers modeled gene expression data using random Boolean networks, mutual information, and probabilistic models to reconstruct regulatory networks.^1^–^18^ These approaches, although useful, provide only indirect evidence of regulatory interactions. Gene perturbation experiments (e.g. transcription factor (TF) knock-out) and ChIP-chip experiments serve as complementary data sources. Gene perturbation experiments uncover functional relations between TFs and their target genes, but they cannot distinguish those indirect relations from direct ones. Hu et al profiled expression with individual deletions of 263 transcription factors in *S. cerevisiae* and used directed-weighted graph modeling and regulatory epistasis analysis to remove indirect regulatory relationships.^19^ ChIP-chip experiments provide direct physical information of the binding between TFs and DNA regions. However, ChIP-chip binding data may not be functional in terms of transcriptional regulation. Most importantly, both types of data are insufficient independently, and depending on the chosen P-value threshold, include many false positive or false negative TF-target relationships.

Since each data source provides partial but complementary information, some research has attempted to integrate those diverse data sources for regulatory network reconstruction.^20^–^37^ A typical approach is to first find potential co-regulated genes and the genes that are further analyzed for other biological evidence, such as common binding motifs and common Gene Ontology (GO) terms. Bar-Joseph et al^24^ relaxed the ChIP-chip P-value threshold if there was strong evidence from expression data. Harbison et al^26^ combined ChIP-chip data, six motif-discovering algorithms, and phylogenetic conservation to construct an initial map of yeast’s transcriptional regulatory code. Lemmens et al^30^ integrated three independent data sources: ChIP-chip data, motif information, and gene expression profiles to correlate regulatory programs with regulators and corresponding motifs to a set of co-expressed genes.

Here we present a novel method to infer relations between TF and target genes by integrating the TF knock-out data and ChIP-chip binding data. Since TF knock-out data suggest functional relations, while ChIP-chip binding data provide physical interactions, the intersection of these two types of data shows strong evidence about transcriptional relations between TF and target genes. However, Hu et al^19^ found that the overlap is quite low, which may be caused by the low quality of the data and the stringent and arbitrary P-value threshold (p < = 0.001). In order to increase the intersection with less false positives, we range both of the P-value thresholds from 0.001 to 0.05 and try to find the optimal P-value threshold pair, at which the most significant intersection is obtained. We demonstrate the method on the yeast data, where it shows that the intersection increases quite a lot. Most inferred TF-target relations have experimental evidence or other computational evidence, which is inferred by combining ChIP-chip data, phylogenetic conservation, motif discovery, other expression data, and enrichment for genes in the same Gene Ontology. The method could be easily extended to identify cooperativity among transcription factors or combine other heterogeneous high-throughput data.

## Methods

**Figure 1.**
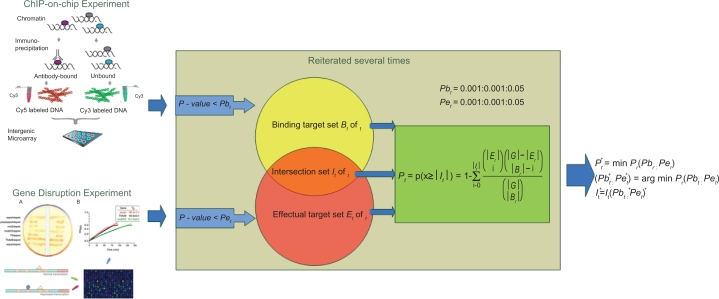
Schematic diagram of the method. The starting point for this method depends on ChIP binding data and TF knockout data (the data sources showed on the left). For each TF, two thresholds are selected for the ChIP binding data and TF deletion data, respectively. When the binding P value of a single gene is less than the binding threshold, this gene is considered to be the binding target. Similarly, if the effectual P value of a single gene in a deletion experiment is less than its assigned threshold, then this gene is defined as the affected target. Both of the two thresholds are set in the range from 0.001 to 0.05 with an increment of 0.001. A value called overlapping significance is calculated based on the binding target set, the affected target set and the intersection of them (the intersecting ovals in the middle). This process is reiterated for all possible combinations of thresholds so that the maximal overlapping significance is obtained (procedures and formulas are showed on the right).

We integrated the ChIP-chip binding data from Harbison et al^26^ and the TF knock out expression data from Hu et al^19^ The overlap of these two data was low at stringent P-value thresholds (both of the P-value < = 0.001). While using lenient P-value thresholds might well improve the overlap, it might also produce many false positives. In order to improve the overlap with less false positives, we ranged both of the P-value thresholds from 0.001 to 0.05 in steps of 0.001, and tried to find for each TF the optimal P-value threshold pair, at which the most significant intersection would be obtained. The schematic diagram of the method is shown in Figure 1.

### Selecting sets of target genes

Let us denote by *G* the common pool of genes that ChIP-chip and knock-out experiments used. Considering a specific transcription factor *t*, we identify two subsets of *G*, binding target set *B_t_* and effectual target set *E_t_*. *B_t_* includes genes with significant ChIP-chip binding to TF *t* (binding P-value < *Pb_t_*), while *E_t_* contains the genes whose mRNA expression are significantly altered in the transcription factor *t* knockout experiments (P-value < *Pe_t_*). *Pb_t_* and *Pe_t_* are P-value thresholds for binding and knock-out experiments respectively. Finally we define the intersection of these two sets *B_t_* and *E_t_* as *I_t_* = *B_t_* ⋒ *E_t_*.

### Calculating the significance of the intersection

To statistically access the significance of the intersection of the two target sets, we calculate the probability of obtaining an intersection size |*I_t_*| this large or greater, given the two sets are independent. With the assumption that *I_t_* is randomly picked, the size of the intersection |*I_t_*| is distributed according to the hypergeometric distribution. The probability to obtain an intersection size |*I_t_*| is computed by the formula, where x represents the random variable for the intersection of two target sets.

$$p(x=|I_{t}|)=\frac{\left(\begin{array}{c} \left|E_{t}\right|\\ \left|I_{t}\right| \end{array}\right)\left(\begin{array}{c} \left|G|-|E_{t}\right|\\ \left|B_{t}|-|I_{t}\right| \end{array}\right)}{\left(\begin{array}{c} \left|G\right|\\ \left|B_{t}\right| \end{array}\right)}$$

The P-value *P_t_* as the probability of observing an intersection this large or greater can thus be computed by the formula, where *x* represents the random variable for the intersection of two target sets.

$$P_{t}=p(x\geq |I_{t}|)=1-\sum_{i=0}^{\left|I_{t}\right|}\frac{\left(\begin{array}{c} \left|E_{t}\right|\\ i \end{array}\right)\left(\begin{array}{c} \left|G|-|E_{t}\right|\\ |B_{t}|-i \end{array}\right)}{\left(\begin{array}{c} \left|G\right|\\ \left|B_{t}\right| \end{array}\right)}$$

### Searching the optimal P-value threshold pair

For each transcription factor *t*, we consider all possible combinations of *Pb_t_* and *Pe_t_* on a scale ranging from 0.001 to 0.05 by an increment of 0.001. The significance of the intersection for each combination is obtained as *P_t_*(*Pb_t_*, *Pe_t_*). Finally, we compare all 2500 (50 × 50) combinations and find the minimum one P_t_*, which is the most significant. The corresponding P-value thresholds are considered to be the optimal pair (Pb_t_*, Pe_t_*). The intersection for choosing the optimal threshold pair, I_t_* is more likely to be the truly target set of the transcription factor *t*.

$$\begin{array}{l} P_{t}^{*}=\text{min}\;P_{t}(Pb_{t},Pe_{t}),\;\;\;Pb_{t}=0.001:0.001:0.05,\\ Pe_{t}=0.001:0.001:0.05,\\ \left(Pb_{t}^{*},Pe_{t}^{*})=\text{arg}\;\text{min}\;P_{t}(Pb_{t},Pe_{t}\right)\\ I_{t}^{*}=I_{\text{t}}(Pb_{t}^{*},Pe_{t}^{*}) \end{array}$$

## Results

The first 30 transcription factors with statistically significant (P*_t_ < 1*e* − 4) intersection between the binding target set and the effectual target set were chosen for further analysis. Overall, 631 unique TF-target gene interactions have been identified using our method, containing 5971 genes regulated by those 30 transcription factors. On the other hand, 430 of the TF-target gene interactions (430/631 = 68.15%) would not be detected if we selected the traditional stringent P-value threshold (both of the P-value < = 0.001). The targets, the optimal P-value threshold pair, and the intersection significance for all TFs are shown in Supplementary Table 1.

### Comparison with YEASTRACT database

**Figure 2.**
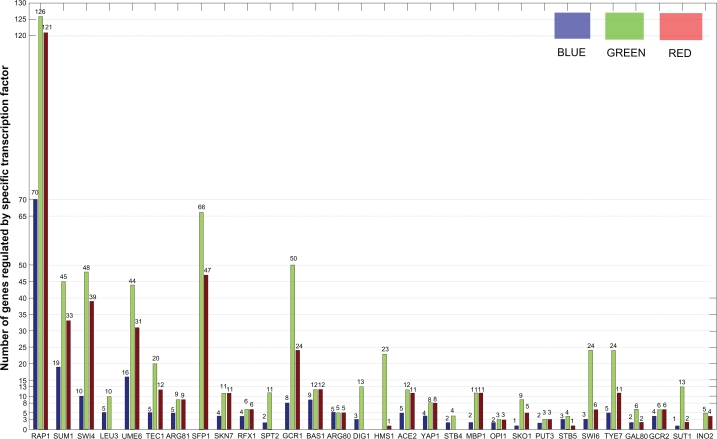
Comparison with YEASTARCT. For the 30 TFs, number of the target genes identified with the stringent P-value threshold pair (*Pb_t_* = 0.001, *Pe_t_* = 0.001) (blue), number of the target genes inferred with the optimal threshold pair (Pb*_t_, Pe*_t_) by our method (green), and the number of our predictions supported in YEASTARCT are shown (red).

YEASTRACT database presently contains regulatory associations of the yeast genes based on more than 1000 bibliographic references.^38^,^39^ To validate our results, we compared the targets identified in our method with documented associations between a Transcription Factor and a target gene in YEASTRACT, which are supported by published data showing at least one of the experimental evidences. As a result, 440 out of the 631 associations in our results have been confirmed. (Those relations found in YEASTARCT are shown in supplementary Table 2). The number of identified targets with stringent P-value cutoff in comparison to that using our method has been shown in Figure 2. The results show that our method significantly reduces the false negatives with less false positives. As an example, RAP1 was assigned to a set of 126 regulated genes using our method, while only 70 targets were identified with stringent P-value cutoff. Out of the remaining 56 targets with our method we found other experimental evidence in YEASTRACT for 51.

### Comparison with high-quality ChIP-chip data

**Figure 3.**
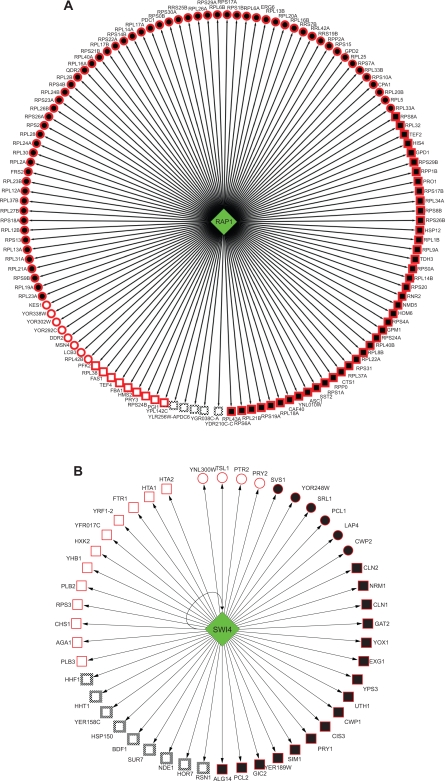
Comparison with high-quality ChIP-chip data. Oval nodes are for genes identified with stringent P-value cutoffs (*Pb_t_* = 0.001, *Pe_t_* = 0.001), while rectangular nodes are for additional genes identified using optimal relaxed threshold pair by our method. Nodes with red solid border are for relations supported by YEASTRACT, otherwise with black dash border. Solid nodes are for the genes supported by high-quality ChIP-chip data. **A**) 126 identified target genes of RAP1. 56 additional target genes are identified (rectangular), while 51 (rectangular with red solid border) are supported by YEASTRACT and 34 (solid rectangular) are supported by high-quality ChIP-chip data. **B**) We have identified 48 target genes of SWI4 including SWI4 itself. SWI4-SWI4 self-regulation is showed by the arrow pointed back to SWI4 itself in the figure. Among SWI4 and other 37 additional target genes identified using optimal relaxed threshold pair by our method (rectangular), as many as 28 (rectangular with red solid border) and SWI4 are supported by YEASTRACT; 16 (solid rectangular) and SWI4 are supported by high-quality ChIP-chip data.

Hu et al^19^ found that the overlap between the binding target set and the effectual target set improved when using the different high-quality ChIP-chip data, suggesting that data quality may be one reason for the low overlap. Our results indicated that the stringent P-value cutoff may be another reason. Even with the low-quality ChIP-chip data, our method obtained 126 common targets for RAP1 between the binding targets and effectual ones, compared with 144 shared between the binding targets from high-quality ChIP-chip and effectual ones. However, out of the 126 targets we found other experimental evidence in YEASTRACT for 121. Furthermore, 104 out of the 126 targets were proven with high-quality ChIP-chip data. In contrast, although only 70 RAP1 targets can be identified at the 0.001 P-value cutoffs, there are still 8 of them not proven. These results indicate that we have reduced 42 false negatives by using relaxed P-value for binding data at the expense of increasing 14 “false positives” even if the high-quality ChIP-chip data are treated as gold standard dataset. However, out of these 14 “false positives” we have found other experimental evidence in YEASTRACT for 9 (see Fig. 3A).

We compared our results with SWI4 high-quality ChIP-chip data (see Fig. 3B), which also suggests that our method can obtain more reliable relations even with the low-quality ChIP-chip data. Only 10 were in the intersection of the binding targets set from low-quality ChIP-chip data and effectual targets set with stringent P-value cutoffs. Also only 16 appeared in the intersection of the binding targets set from high-quality ChIP-chip data and effectual targets set. However, 48 were detected using a pair of relaxed optimal P-value cutoffs (0.04 for the binding P-value and 0.029 for the effectual P-value) even with the low-quality ChIP-chip data. 23 out of the 48 targets are proven with high-quality ChIP-chip data. Additionally, 39 out of the 48 targets have been confirmed in YEASTRACT. Out of the 9 remaining targets (HHF1 HHT1 YER158C, HSP150, BDF1, SUR7, NDE1, HOR7, RSN1) for which we cannot find evidence in YEASTARCT, HHT1 and HHF1 are histone genes. Whole-genome binding studies have suggested that the histone gene promoters are bound by MBF and/or SBF^40^,^41^ and Hess et al’s data^42^ showed that swi4Δ causes a mild reduction in HHT1and HHF1 mRNA levels. Furthermore MBF (Mbp1 and Swi6) and SBF (Swi4 and Swi6) cause transcriptional defects at HTA1-HTB1 and HHT1-HHF1.^42^ Inferred from the above information, HHT1 and HHF1 may be the novel targets of SWI4. Reinoso-Martín^43^ found that HSP150 mRNA levels were slightly induced by caspofungin after 1 hour in wild-type cells but increased significantly in the swi4Δ mutant, which suggests that HSP150 is one target of SWI4.

### Overlap with literature

Our results have well coincided with previous biological literature. As an example, consider Leu3, a pathway-specific regulator of genes encoding enzymes involved in branched-chain amino acid biosynthesis. Using our methods, we have found that LEU3 regulates 5 additional genes (LEU4, ILV5, ILV3, ALD5 and ISU2) that would not have been identified using the stringent 0.001 P-value threshold pair. Two of them (LEU4 and ILV5) are among the seven established LEU3 targets that comprise the pathway for branched amino acid biosynthesis.^44^ Three of these genes (LEU4, ILV5 and ILV3) have been annotated as being involved in “branched chain family amino acid biosynthesis”. Furthermore, the other two genes (ALD5 and ISU2) have been inferred as Leu3 targets using computational methods combining ChIP and expression analyses.^45^

As another example, consider GCR1, which is required for maximal transcription of many genes, including genes encoding glycolytic enzymes. Tpi1p is an abundant glycolytic enzyme that makes up about 2% of the soluble cellular protein while GCR1 binding is required for activation of TPI1.^46^ Other glycolytic genes such as ENO2 and ADH1 are dependent on GCR1 gene function for full expression.^47^,^48^ Finally, consider transcription factors that have functions previously reported to control the cell cycle during growth. The UME6 gene of *S. cerevisiae* was identified as a mitotic repressor of early meiosis-specific gene expression. It provides target specificity by binding to the URS1 sequence element (TAGCCGCCGA) that is located upstream from many early meiosis-specific genes. UME6 (“Unscheduled Meiotic gene Expression”) is a key transcriptional regulator of early meiotic genes such as SPO1^49^,^50^ and SPO13. 49–51 In addition to the regulation of meiosis-specific genes, UME6 has been implicated in the transcriptional regulation of genes involved in arginine catabolism. Expression of the catabolic genes CAR1 encoding arginase and omithine transaminase is repressed by nitrogen. Previous studies have indicated that the UME6 gene is involved in mediating this repression.^51^,^52^

To further validate our results, we selected some transcriptional factors whose target genes prediction showed a relatively low overlap with information from YEASTRACT, and compared them with other predictions of MacIsaac KD et al^53^ and Pham TH et al^54^ MacIsaac KD et al^53^ combined phylogenetic conservation-based motif discovery algorithms, PhyloCon, and Converge to create a refined regulatory map for *S. cerevisiae* by reanalyzing the same ChIP-chip binding data. Pham TH et al^54^ developed a method that combined three different expression datasets with the same ChIP-chip binding data with a relaxed threshold (P-value = 0.005) to discover target genes based on rule induction. Although our methods combined data TF knock-out data different than MacIsaac KD et al^53^ and Pham TH et al^54^ and used a different approach, the results showed that most of our predictions that were not supported by YEASTARCT could be proven by data from MacIsaac KD et al^53^ and Pham TH et al.^54^ For example, our method identified 21 additional targets of SWI6 with the optimal relaxed P-value thresholds pair. Unfortunately we could find evidence from YEASTRACT for only 5 of them. However, 13 of the 21 additional targets were also predicted by the study of MacIsaac KD et al^53^ and 9 of them were inferred by the study of Pham TH et al^54^ Combining the evidence from the above two sources and information from YEASTRACT, 14 in 21 have been convinced of genuine targets of SWI6 (see Table 1). Among the left 7 target genes, YMR144 W and YOR248 W were predicted as SWI6 targets by Harbison et al^26^ Other five genes (CIS3, YER079 W, FTR1, PLB3, and HTZ1) could be inferred as SWI6 targets as they showed close relationship with the SBF complex (SWI4/SWI6). CIS3, a glycoprotein-encoding gene, was reported to have conserved binding sites for SWI6-SWI4 complex.^55^ YER079 W, FTR1, PLB3, and HTZ1 also showed evidence to be related with SWI4.^55^ As another example, although all of the 10 additional targets of DIG1 could not be supported by YEASTRACT, 6 targets could be found in the results of MacIsaac KD et al^53^ and 5 in Pham TH et al^54^ (see Table 2). In the remaining 4 genes, MFA1 and AGA2 were involved in mating or pheromone response;^56^ they stood a good chance to be the targets of DIG1, which was also known to be involved in the regulation of mating-specific genes and the invasive growth pathway.^57^

### Gene ontology enrichment analysis

Finally, to ensure that we found biologically meaningful targets, we performed Gene ontology analyses using the Saccharomyces Genome Database web site to evaluate whether a gene set was enriched for biologically relevant targets (see Table 3). It turned out that the regulated gene sets generally identified groups of genes that functioned in a similar biological pathway and were generally accurate in assigning regulators to sets of genes whose functions were consistent with the regulators’ known roles. For example, ARG80 was well known to be a transcription factor required for specific regulation of arginine metabolism in yeast.^58^ Four out of the five genes (P-value ≤ 3e-15) (ARG5,6/YER069W, ARG3/YJL088 W, ARG8/YOL140W, CPA1/YOR303W) that we identified using our method were annotated as being involved in “arginine biosynthetic process”. The same situation happened to GAL80, which was a well-characterized transcription factor involved in a genetic switch. The switch, which consisted of three proteins, controlled the genes that encoded the enzymes required for galactose metabolism at the level of transcription.^59^ Four out of six genes that we identified as the targets of GAL80 (GAL7/YBR018C, GAL10/YBR019C, GAL1/YBR020W, GAL2/YLR081W) were involved in galactose metabolic process. For another example, GCR2 was the transcription factor affecting expression of most glycolytic genes in *S. cerevisiae.*^60^ All six of these genes were directly on the committed pathway to leucine or valine biosynthesis (PGI1/YBR196C, TPI1/YDR050C, TDH3/YGR192C, TDH2/YJR009C, FBA1/YKL060C, and GPM1/YKL152C).

## Discussion

ChIP-chip data contain information about physically binding interactions, while TF knock-out experiments provide information about functional relations. By combining these two complementary data sources, the method is expected to uncover the TF-target relations. However, the data quality and the arbitrary P-value threshold lead to the low overlap between these two data. In this study, we developed a novel method to integrate these two data for inferring TF-target gene relations. The key aspect of our approach is to find the optimal P-value threshold pair for each TF, at which the most significant overlap is obtained. Our method is powerful because it allows the P-value threshold to be relaxed if there is supporting evidence from each of these two complementary data. Comparison of the results with the YEASTRACT and the literature shows that experimental evidence exists for most of TF-target gene relations in our results. Considering those relations between TF and target genes for which there is no direct experimental evidence, we are able to found other computational evidence. Furthermore a plausible explanation could often be inferred from the functional links between the TF and target genes.

It should be noted that although we focused on the TF-target gene relations, our method could be easily extended to discover the cooperativity among transcription factors by combining these two data from different TFs. It could also be used to combine the information from multiple ChIP-chip experiments on the same TF when these data are available. With more and more genomic data available, it will become an inevitable trend to study the complex biological systems based on computational integration of those heterogeneous data. Our work provides a simple but novel method to integrate available biological information in a principled fashion.

## Figures and Tables

**Table 1.** List of SWI6 targets with computational evidence.

**TFs**	**ORF**	**YEASTRACT**	**MacIsaac KD et al^53^**	**Pham TH et al^54^**	**Literature evidence**
SWI6	YBR071W		x		x
SWI6	CHA1			x	x
SWI6	HTA1		x	x	x
SWI6	YER079W				
SWI6	PUP3		x	x	x
SWI6	SWI4		x		x
SWI6	FTR1				
SWI6	CIS3				
SWI6	RPS4A		x		x
SWI6	HMS2		x		x
SWI6	CWP2	x	x		x
SWI6	EXG1		x	x	x
SWI6	YOX1	x	x	x	x
SWI6	YMR144W				
SWI6	SCW10	x	x	x	x
SWI6	PLB3				
SWI6	HTZ1				
SWI6	SKM1		x	x	x
SWI6	SRL1	x	x	x	x
SWI6	YOR248W				
SWI6	OPY2	x	x	x	x

**Table 2.** List of DIG1 targets with computational evidence.

**TFs**	**ORF**	**YEASTRACT**	**MacIsaac KD et al^53^**	**Pham TH et al^54^**	**Literature evidence**
DIG1	UBC4		x	x	x
DIG1	TEC1		x		x
DIG1	KAR4		x	x	x
DIG1	YDR042C				
DIG1	YDR210C-D				
DIG1	MFA1				
DIG1	STE2		x	x	x
DIG1	AGA2				
DIG1	BAR1		x	x	x
DIG1	ARO7		x	x	x

The notion ‘X’ denotes “overlapped results”. The last column combines the left three columns, indicating whether there is any evidence from YEASTRACT, MacIsaac KD et al^53^ and Pham TH et al.^54^

**Table 3.** List of some enriched GO annotations.

**Regulators**	**Functional description of regulators**	**# of genes**	**Significantly shared GO annotations**	***P* value**
**RAP1**	High level transcriptional activation of genes encoding ribosomal proteins and glycolytic enzymes	126	(86/126) structural constituent of ribosome	2.66E–100
			(91/126) translation	8.79E–83
**SUM1**	Mitotic repression of middle sporulation-specific genes, general replication initiation	45	(16/45) sporulation	9.93E–15
**LEU3**	Regulates the transcription of genes encoding enzymes involved in branched-chain amino acid synthesis	10	(6/10) branched chain family amino acid biosynthetic process	1.45E-13
**UME6**	Transcriptional regulator of early meiotic genes, transcriptional regulation of genes involved in arginine catabolism	44	(2/43) arginine catabolic process	0.00571
			(7/43) meiosis	0.00797
**TEC1**	Required for full Ty1 expression, Ty1-mediated gene activation	20	(17/20) transposition, RNA-mediated	3.85E-25
**ARG81**	Involved in the regulation of arginine-responsive genes	9	(6/9) arginine metabolic process	2.78E-13
**SFP1**	Controls expression of many ribosome biogenesis genes in response to nutrients and stress, regulates G2/M transitions during mitotic cell cycle and DNA-damage response	66	(42/66) structural constituent of ribosome	8.62E-45
			(46/66) translation	2.90E-39
**RFX1**	Involved in DNA damage and replication checkpoint pathway	6	(3/6) deoxyribonucleotide biosynthetic process	2.18E-07
**GCR1**	Transcriptional activators of glycolytic genes	50	(10/50) glycolysis	5.94E-14
**BAS1**	Involved in the expression of genes encoding enzymes acting in the histidine, purine, and pyrimidine biosynthetic pathways	12	(4/12) purine ribonucleoside monophosphate biosynthetic process	1.28E-07
**ARG80**	Involved in regulation of arginine-responsive genes	5	(4/5) arginine biosynthetic process	7.63E-10
**DIG1**	Involved in the regulation of mating-specific genes, inhibits pheromone-responsive transcription	13	(8/13) sexual reproduction	1.89E-09
			(8/13) response to pheromone	2.93E-10
**HMS1**	Overexpression confers hyperfilamentous growth	23	(15/23) cytosolic part	1.02E-15
**ACE2**	Activates transcription of genes expressed in the G1 phase	12	(4/12) cytokinesis, completion of separation	8.06E-08
**YAP1**	Activates the transcription of anti-oxidant genes in response to oxidative stress	8	(4/8) response to oxidative stress	5.32E-05
**OPI1**	Negative regulation of phospholipid biosynthetic genes	3	(2/3) fatty acid synthase complex	2.54E-06
**SKO1**	Cytosolic and nuclear protein involved in osmotic and oxidative stress responses	9	(2/9) structural constituent of cell wall	0.00094
**TYE7**	transcriptional activator in Ty1-mediated gene expression, binds E-boxes of glycolytic genes and contributes to their activation	24	(9/24) transposition, RNA-mediated	7.74E-08
			(4/24) glycolysis	9.21E-05
**GAL80**	involved in transcriptional regulation in response to galactose	6	(4/6) galactose metabolic process	1.86E-09
**GCR2**	transcriptional activators of glycolytic genes	6	(6/6) glycolysis	5.08E-14
**SUT1**	involved in sterol uptake; involved in induction of hypoxic gene expression	13	(3/13) structural constituent of cell wall	1.37E-05
**INO2**	required for derepression of phospholipid biosynthetic genes in response to inositol depletion	5	(4/5) lipid biosynthetic process	2.77E-05

Functional description of regulators is from the Saccharomyces Genome Database.

Gene Ontology analysis done using GO Term Finder in SGD in Aug 31, 2008; 5952 genes were included in the background set with P-value cut-off < 0.01.
